# Machine learning approach for predicting the severity risk of obstructive sleep apnea syndrome

**DOI:** 10.3389/fdata.2026.1832790

**Published:** 2026-07-16

**Authors:** Qi Wang, Xiaoyu Yang, Shuran Xu, Haohao Wu, Guixuan Wang, Huixian Liu, Ronghua Chen, Fengming Xu, Cheng Wang, Kang Du

**Affiliations:** 1 Department of Neurology, Xuanwu Hospital, Capital Medical University, Beijing, China; 2 Department of Neurology, Affiliated Qujing Hospital of Kunming Medical University/Qujing Central Hospital of Yunnan Province, Qujing, China; 3 Computer Innovation Technology Research Institute of Zhejiang University, Zhejiang, Hangzhou, China

**Keywords:** apnea-hypopnea index, artificial intelligence, machine learning, obstructive sleep apnea hypopnea syndrome, prediction, XGBoost

## Abstract

**Background:**

Obstructive Sleep Apnea-Hypopnea Syndrome (OSAHS) has a high global prevalence and is prone to causing various serious complications. Our objective is to develop severity stratification of OSAHS by integrating multiple commonly available clinical features based on machine learning (ML).

**Materials and methods:**

This study collected data from 432 cases at Qujing Central Hospital in Yunnan Province, integrating 25 clinical feature variables. The cases were randomly split into training (70%) and validation (30%) sets. The importance of the 25 features was analyzed.

**Results:**

It showed that the HCY, TBIL, BMI, GGT, and Age made significant contributions to OSAHS severity. We established five machine learning models-Multilayer Perceptron (MLP), Random Forest, XGBoost, LightGBM, and Support Vector Machine (SVM)-by integrating 25 clinical features. Through cross-validation and continuous adjustment of model parameters, the optimal predictive model was determined. By calculating model accuracy and *F*1-score, XGBoost was identified as the best-performing model, achieving an area under the curve (AUC) of 0.63, an accuracy of 75% and an *F*1-score of 65.60.

**Conclusion:**

In this study, we established a predictive model for the severity stratification of OSAHS based on machine learning algorithms. The XGBoost model demonstrated superior predictive performance.

## Introduction

Obstructive Sleep Apnea Hypopnea Syndrome (OSAHS) has a high global prevalence, with approximately 425 million individuals affected by moderate to severe disease. Clinically, the Apnea-Hypopnea Index (AHI) serves as the basis for grading disease severity, as shown in Table 1, where an AHI of ≥15 events per hour indicates moderate to severe patients. However, due to factors such as insufficient public awareness and uneven distribution of medical resources, the actual diagnosis rate of this condition remains below 1% (Peppard et al., 2013; Benjafield et al., 2019). OSAHS is associated with numerous risk factors, including obesity, male gender, aging, upper airway anatomical abnormalities, family history, and unhealthy lifestyles (such as chronic alcohol consumption and smoking) (Xu and Jin, 2025). It is prone to causing complications such as hypertension, coronary heart disease, type 2 diabetes mellitus, stroke, and cognitive impairment, severely impacting social productivity and patient quality of life (Kariuki et al., 2022; Yuan et al., 2021; Gao et al., 2023). Polysomnography (PSG) is the “gold standard” for diagnosing OSAHS. It comprehensively evaluates sleep structure and respiratory events by synchronously recording multiple physiological signals (Chinese Medical Association et al., 2019). However, issues such as high cost, complex operation, and low accessibility have limited its widespread application, leading to diagnostic delays (Abad et al., 2016). Therefore, developing an auxiliary tool capable of rapidly and accurately stratifying OSAHS severity holds significant clinical value for optimizing healthcare resource allocation and enabling precise diagnosis and treatment.

**Table 1:** Severity classification of adult obstructive sleep apnea-hypopnea syndrome (OSAHS).

Severity classification	Mild	Moderate	Severe
AHI (events/h)	5–15	15–30	>30
Minimum oxygen saturation	85–90	80–85	< 80

AHI, apnea-hypopnea index. Although clinical severity is traditionally classified into three grades, for the purpose of this prediction model, we simplified it into a binary outcome: mild (AHI ≤ 15) and moderate-to-severe (AHI > 15).

In recent years, breakthroughs in artificial intelligence (AI) and machine learning (ML) technologies have provided new opportunities for addressing the aforementioned challenges (Sim et al., 2023; Handelman et al., 2018; Swanson et al., 2023). Studies have demonstrated that high-performance predictive models for OSAHS can be constructed by integrating patient demographic information (such as age, gender, and BMI), clinical symptoms (e.g., snoring, daytime sleepiness), and simple questionnaire scores (e.g., the Epworth Sleepiness Scale) (Huang et al., 2024). Unlike previous studies that primarily relied on demographic and symptom data, our work emphasizes the integration of biochemical markers (specifically HCY and TBIL) with routine clinical indicators, providing a more holistic and accessible approach for OSAHS severity prediction. This study aims to utilize the powerful data modeling capabilities of machine learning to develop a predictive model capable of rapidly stratifying the severity of OSAHS. We integrated 25 clinical indicators, including BMI, red blood cells, platelets, blood pressure, and blood glucose, as input variables for the machine learning model, striving to create a model that can quickly achieve rapid severity stratification for OSAHS. It is hoped that this model can replace or assist PSG in diagnosis to optimize medical resources and reduce the disease burden of OSAHS (Han and Oh, 2023).

## Materials and methods

### Study population and data collection

This study utilized data from 432 patients at Qujing Central Hospital in Yunnan Province. All participants underwent assessments of 25 clinical indicators, including blood glucose, BMI, age, PSG, and carotid ultrasound. Missing data for numerical features were imputed using the median value, which is robust to outliers and consistent with standard practice for small clinical datasets. The AHI was determined through PSG monitoring, and each patient was classified according to OSAHS severity. The task was formulated as a binary classification: Class 0 (Mild OSA, AHI ≤ 15) and Class 1 (Moderate-to-Severe OSA, AHI > 15). Patients with an AHI > 15 were categorized as having moderate-to-severe OSAHS, while those with an AHI ≤ 15 were classified as mild cases. The study data were obtained from medical records of Qujing Central Hospital in Yunnan Province. A total of 432 patients were included. All participants provided informed consent; were aged ≥18 years; had not received any prior treatment for OSAHS; reported nocturnal snoring; and underwent PSG, carotid ultrasound, and blood tests. Patients were excluded if they did not provide informed consent; were aged < 18 years; lacked snoring symptoms; had a history of uremia, kidney or heart transplantation, severe heart failure, or multiple organ dysfunction; had incomplete baseline data; or were pregnant. Patient data are provided in the supplementary materials. The dataset was randomly split into a training set (70%) and an independent test set (30%). The dataset has a natural class distribution (Mild: 31.7%, Moderate-Severe: 68.3%). We used stratified sampling for the train-test split to preserve this distribution. Importantly, the dataset partitioning was performed strictly at the patient level, ensuring that no single patient appeared in both the training and test sets, thereby preventing data leakage and better reflecting real-world clinical application.

### Dataset description

A total of 25 clinical data indicators were collected from patients, comprising 25 candidate variables. The indicators are as follows: Gender: This feature refers to a person's gender. Age (years): This feature refers to the age of a person who is over 18 years old. It is numerical data. The number of men is 360 (83.33%), while the number of women is 72 (16.67%). It is nominal data. BMI (kg/m^2^): This feature is calculated as weight/height (Benjafield et al., 2019). It is numerical data. RBC (**10**^**12**^/L): This feature refers to the count of red blood cells in the blood. It is numerical data. PLT (10^9^/L): This feature refers to the count of platelets in the blood. It is numerical data. MPV (fl): This feature refers to the mean volume size of platelets in the blood. It is numerical data. PDW (fl): This feature refers to the dispersion of platelet volume size in the blood. It is numerical data. HB (g/L): This feature refers to the mass of hemoglobin per unit volume of blood. It is numerical data. HCT (%): This feature refers to the volume of red blood cells per unit volume of blood. It is numerical data. TG (mg/dl): This characteristic refers to the mass of triglycerides per unit volume of blood. It is numerical data. TC (mg/dl): This feature captures the participant's total cholesterol. It is numerical data. HDL-C (mmol/L): This feature captures the participant's high-density lipoprotein. It is numerical data. LDL-C (mmol/L): This feature captures the participant's low-density lipoprotein. It is numerical data. HCY (μmol/L): This feature refers to the plasma concentration of homocysteine. It is numerical data. SBP (mmHg): This feature captures the participant's systolic blood pressure. It is numerical data. DBP (mmHg): This feature captures the participant's diastolic blood pressure. It is numerical data. ALT (U/L): This feature refers to the serum concentration of alanine aminotransferase. It is numerical data. GGT (U/L): This feature refers to the patient's glutamyltranspeptidase. It is numerical data. TBIL (μmol/L): This feature refers to the patient's total bilirubin. It is numerical data. Cr (μmol/L): This feature refers to the patient's creatinine. UA (μmol/L): This characteristic refers to the patient's uric acid. It is numerical data. Glucose (mg/dl): This feature refers to the plasma concentration of glucose. It is numerical data. Hypertension (mmHg): This feature refers to the patient's blood pressure in the context of hypertension. It is numerical data. Diabetes (mmol/L): This feature refers to the patient's relevant blood glucose indicator blood glucose. It is numerical data. Common carotid artery plaque: This feature refers to the presence or absence of carotid artery plaque in the patient. It is categorical data.

### Data preprocessing

Numerical features were standardized using z-score normalization (mean = 0, *SD* = 1) prior to model training. Categorical features (e.g., gender, carotid plaque) were one-hot encoded.

### Features ranking

To ensure transparency and reproducibility, the feature selection process was restructured into a clear, three-step workflow: first, redundant features were removed using Pearson correlation coefficients, excluding features with a correlation coefficient |*r*| ≥ 0.9 to avoid multicollinearity. Second, Information-based ranking using Information Gain and Gain Ratio to evaluate the discriminative power of each feature. Finally, random forest feature importance was used for stability verification. The Pearson correlation coefficient was used to quantify the linear association strength between variables and visualized as a correlation heatmap using Origin software (Cao et al., 2025). The color gradient (e.g., from blue to red) intuitively displays the pairwise correlation coefficients, facilitating rapid identification of strong variable associations. We also evaluated the importance of the 24 features using Information Gain and assessed inter-feature correlations using the Gain Ratio (Epstein et al., 2023). Subsequently, feature ranking was performed to select the features with the optimal Gain Ratio. Additionally, we employed the Random Forest function from the scikit-learn library to rank the features based on their importance (Speiser, 2021).

### Machine learning models

A total of 432 patients were divided into two groups: a training set and a test set. The training set was used to establish models for predicting the severity of OSAHS patients, while the test set was used to validate the models. Five machine learning algorithms—Multilayer Perceptron (MLP), Random Forest, eXtreme Gradient Boosting (XGBoost), Light Gradient Boosting Machine (LightGBM), and Support Vector Machine (SVM)—were employed to construct the models. The test set was strictly isolated and not involved in model training or hyperparameter tuning, serving as an unbiased evaluation of real-world performance. The area under the curve (AUC) values of the models were calculated to evaluate their predictive accuracy. XGBoost is an ensemble learning technique that builds a powerful predictive model by training multiple decision trees within a gradient boosting framework and combining the outputs of all trees (Ye et al., 2023). The decision tree is constructed by determining the split points based on the prediction errors across all input variables, while additional regression trees are built using the residuals from the previous tree. This sequential prediction approach significantly enhances the model's predictive accuracy (Liu K. et al., 2024). LightGBM is a gradient boosting framework that scans each data point and estimates potential information gain (Han and Oh, 2023). LightGBM enhances model training accuracy and improves training efficiency through its hierarchical learning strategy (Zhu et al., 2025). SVM is a classifier capable of establishing a decision boundary called a hyperplane to optimally separate two distinct classes (Liu K. et al., 2024). The MLP is an artificial neural network composed of an input layer, one or more hidden layers, and an output layer, with full connections between each layer. Currently, MLP is widely used in various clinical research applications and has demonstrated favorable performance (Wu et al., 2022). Random Forest is an ensemble method that creates a combination of decision trees by using randomly selected samples for classification (Han and Oh, 2023).

### Evaluation metrics

To evaluate the predictive performance of the machine learning models, we measured the metrics of accuracy, precision, recall, and *F*1-score. Given the specific distribution of the dataset, model performance was primarily evaluated using Accuracy and the *F*1-score to balance the trade-off between false positives and false negatives (FN). The confusion matrix was used to facilitate a deeper understanding and calculation of these metrics. True Positive (TP): This term refers to an instance where the data's true value is positive and the predicted value is also positive. False Negative (FN): This term refers to an instance where the data's true value is positive, but it is incorrectly predicted as negative. False Positive (FP): This term refers to an instance where the data's true value is negative, but it is incorrectly predicted as positive. True Negative (TN): This term refers to an instance where the data's true value is negative and the predicted value is also negative. Accuracy: It represents the percentage of correctly identified samples out of the total number of samples. Accuracy = (TP + TN)/(TP + FN + FP + TN); Precision: It represents the percentage of samples that are identified as positive and are actually positive. Precision = TP/(FP + TP); Recall: It represents the proportion of actual positive samples that are correctly predicted as positive out of the total positive samples. Recall = TP/(FN + TP); The *F*1 is the weighted average of precision and recall; *F*1-score = 2 ^*^ Precision ^*^ Recall/(Recall + Precision) (Zhao et al., 2023; Begum and Kalaivani, 2025).

### Statistical analysis

The establishment and analysis of all models were implemented using the Python programming language. Data analysis and processing were performed utilizing the Python-based Numpy and Pandas libraries. Statistical analysis of clinical data was conducted using GraphPad Prism (Version 10). We employed GraphPad Prism 10 software to analyze demographic and clinical data, and assessed data normality using the Normality and Lognormality test. For data conforming to a normal distribution, the *t*-test was applied, while the Mann–Whitney *U*-test was used for non-normally distributed data. For binary categorical variables, the Chi-squared test was employed. A *p*-value < 0.05 was considered statistically significant. Meanwhile, we evaluated model performance by combining grid search with cross-validation. Specifically, for each hyperparameter combination, *K*-fold cross-validation was performed: the training set was split into *K* folds, with *K* – 1 folds used for training and the remaining fold for validation in each iteration. The average validation score across the *K* iterations was calculated for that parameter combination. After iterating through all parameter combinations, the set with the highest average score was selected as the optimal hyperparameters.

### Model development and validation

Grid search with 5-fold cross-validation performed exclusively on the training set to select optimal hyperparameters. All reported performance metrics (Accuracy and *F*1-score) in the Results section were derived exclusively from the independent test set. To mitigate overfitting, we constrained model complexity by limiting the maximum tree depth for tree-based models (XGBoost, LightGBM, Random Forest) and incorporated L1/L2 regularization terms into the loss functions.

## Results

### Demographic and clinical characteristics

As summarized in Table 2, significant differences were observed between the mild OSAHS group and the moderate-to-severe OSAHS group in terms of MPV (*P* = 0.027) and DBP (*P* = 0.033). There were no statistically significant differences between the two groups in terms of gender, age, BMI, RBC, PLT, PDW, HB, HCT, TG, TC, HDL-C, LDL-C, HCY, SBP, ALT, GGT, TBIL, Cr, UA, glucose, hypertension, diabetes, or common carotid artery plaque (bilateral/unilateral/non; *P* > 0.05).

**Table 2:** Comparative analysis of demographic characteristics in the training cohort for machine learning models: mild vs. moderate-to-severe obstructive sleep apnea-hypopnea syndrome (OSAHS).

Baseline characteristics	Mild OSAHS (*n* = 137)	Moderate and severe OSAHS (*n* = 295)	*P*-value
Gender (M/F)	111/26	249/46	0.406
Age (year)	51.58 ± 12.60	51.22 ± 12.09	0.776
BMI (kg/m^2^)	27.00 ± 5.80	27.70 ± 4.50	0.072
RBC (10^12^/L)	5.12 ± 0.90	5.24 ± 0.96	0.262
PLT (10^9^/L)	215.00 ± 76.00	219.00 ± 79.50	0.532
MPV (fl)	10.50 ± 1.40	10.30 ± 1.30	0.027^*^
PDW (fl)	12.40 ± 3.62	12.20 ± 3.10	0.328
HB (g/L)	159.00 ± 26.00	161.00 ± 29.00	0.472
HCT (%)	46.80 ± 7.45	47.80 ± 7.80	0.179
TG (mg/dl)	1.96 ± 1.45	1.86 ± 1.53	0.432
TC (mg/dl)	4.72 ± 1.29	4.68 ± 1.37	0.771
HDL-C (mmol/L)	1.05 ± 0.28	1.06 ± 0.26	0.940
LDL-C (mmol/L)	3.00 ± 0.76	3.04 ± 0.74	0.598
HCY (μmol/L)	12.60 ± 7.23	12.50 ± 6.00	0.656
SBP (mmHg)	134.00 ± 28.70	137.00 ± 30.00	0.205
DBP (mmHg)	86.00 ± 20.50	88.00 ± 20.00	0.033^*^
ALT (U/L)	26.00 ± 20.00	29.00 ± 24.00	0.206
GGT (U/L)	38.00 ± 38.00	41.00 ± 36.50	0.375
TBIL (μmol/L)	13.45 ± 8.33	13.60 ± 7.60	0.614
Cr (μmol/L)	74.30 ± 24.00	76.50 ± 24.00	0.254
UA (μmol/L)	399.00 ± 128.00	417.00 ± 133.00	0.239
Glucose (mmol/L)	5.40 ± 1.35	5.50 ± 1.45	0.401
Hypertension	81/55	197/98	0.160
Diabetes	28/108	59/236	0.898
Common carotid artery plaque (bilateral/unilateral/ non)	11/14/37	70/33/70	0.467

BMI, body mass index; RBC, red blood cell; PLT, platelet; MPV, mean platelet volume; PDW, platelet distribution width; HB, hemoglobin; HCT, hematocrit; TG, triglyceride; TC, total cholesterol; HDL-C, high-density lipoprotein cholesterol; LDL-C, low-density lipoprotein cholesterol; HCY, homocysteine; SBP, systolic blood pressure; DBP, diastolic blood pressure; ALT, alanine aminotransferase; GGT, gamma-glutamyl transferase; TBIL, total bilirubin; Cr, creatinine; UA, uric acid.

Of the 25 features compared between the mild OSAHS group and the moderate and severe OSAHS group using student's *t*-test and Chi-square test, normally distributed data are expressed as mean ± standard deviation, and non-normally distributed data as median ± interquartile range. The results revealed significant differences in MPV (*P* = 0.027) and DBP (*P* = 0.033).

^*^P < 0.05.

### Feature ranking

**Figure 1:**
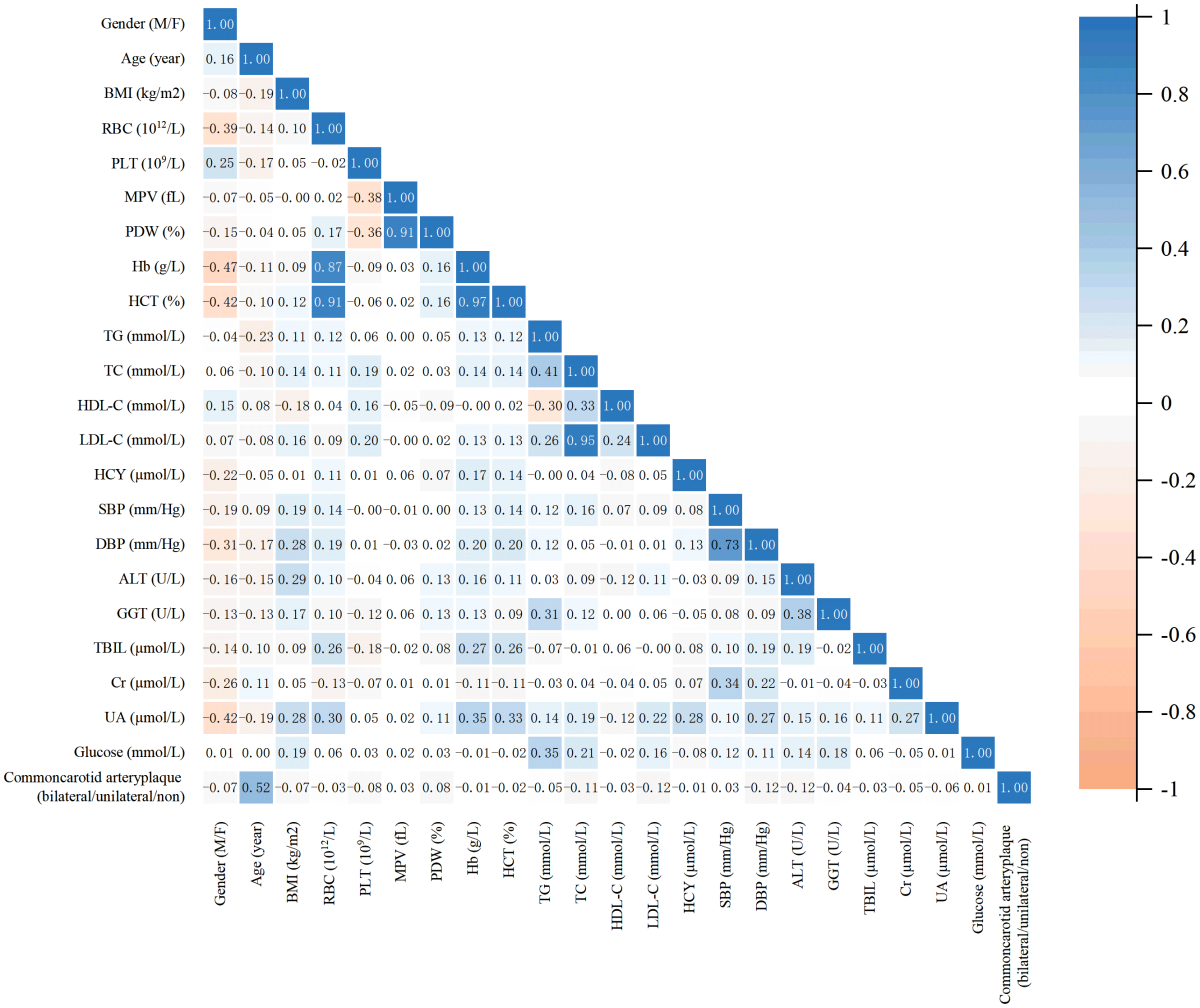
Correlation heatmap between the 25 features.

**Figure 2:**
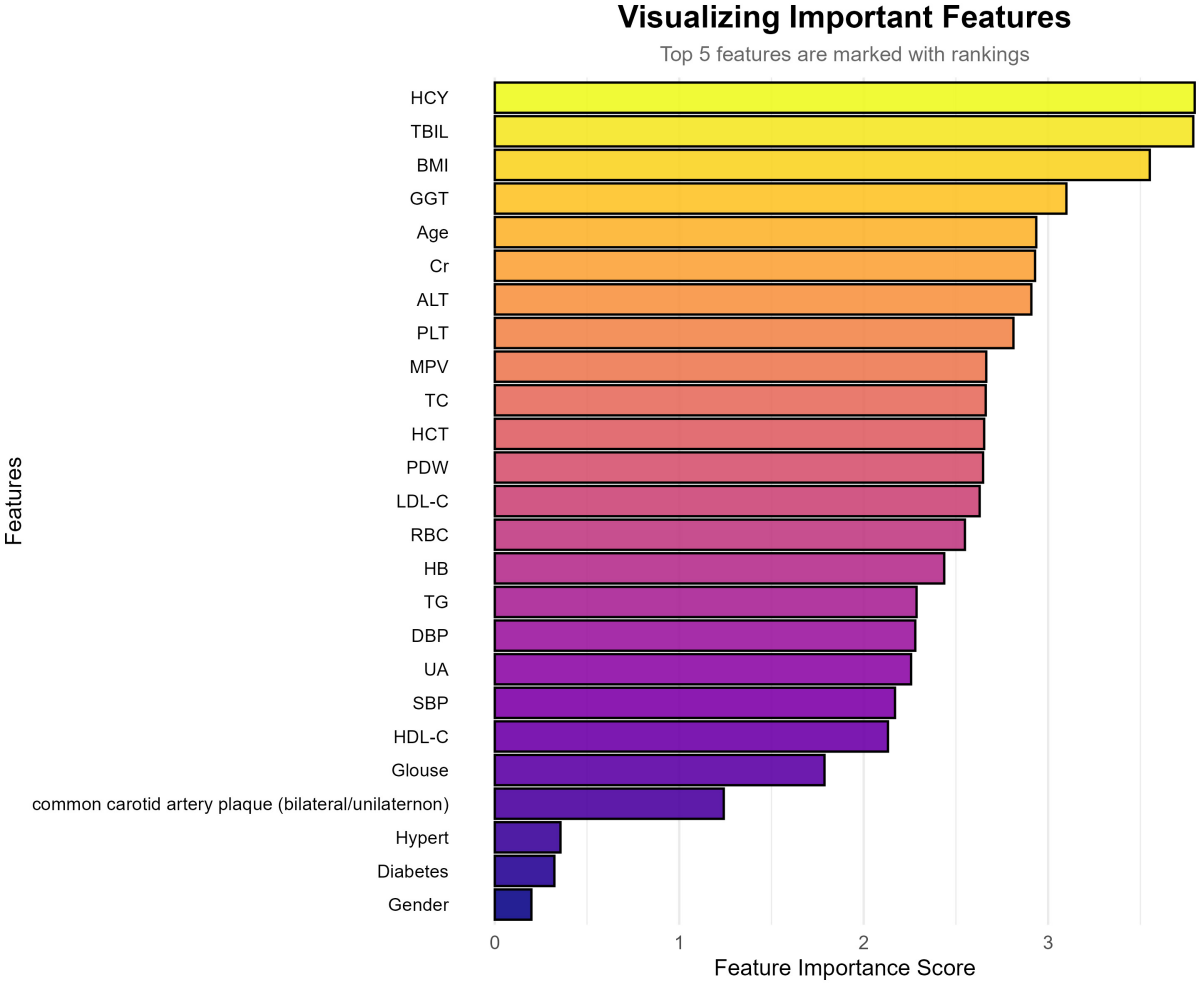
Feature importance ranking of the 25 features.

The Figure 1 presents the results of the correlation analysis in a heatmap. In the correlation plot, we observed strong linear relationships between several feature pairs: HB and HCT demonstrated a very strong positive correlation (*r* = 0.97). HDL-C and LDL-C exhibited a strong positive correlation (*r* = 0.95). PDW and PLT showed a strong positive correlation (*r* = 0.91). SBP and DBP displayed a moderately strong positive correlation (*r* = 0.73). RBC and HB also showed a strong positive correlation (*r* = 0.87). Additionally, TC and LDL-C had a moderate positive correlation (*r* = 0.41), while ALT and GGT displayed a weak-to-moderate positive correlation (*r* = 0.38). Negative correlations were also identified: BMI and RBC had a weak negative correlation (*r* = −0.39), suggesting that higher BMI may be associated with lower RBC count; Cr and RBC showed a weak negative correlation (*r* = −0.26); and UA and RBC demonstrated a moderate negative correlation (*r* = −0.42). Figure 2 presents the prediction contribution gradients of the 25 features. Among them, HCY, TBIL, BMI, GGT, and Age are identified as core predictive indicators, contributing the most to the prediction of OSAHS severity classification. In Table 3, the Pearson rank represents the strength and direction of the linear correlation between the features and the outcome. A larger absolute value indicates a stronger correlation, with positive values indicating a positive correlation and negative values indicating a negative correlation. Statistical significance is considered when *P* < 0.05. InfoGain reflects the information provided for outcome classification, with higher values indicating greater importance. Gain-Ratio corrects for the preference of information gain toward multi-category attributes, offering a fairer measure. Among the 25 indicators, DBP shows the strongest positive correlation with statistical significance, while also demonstrating relatively high information gain and gain-ratio, identifying it as a core risk factor. MPV exhibits a significant negative correlation, suggesting that higher MPV may be associated with lower risk. The indicators with the highest information gain are TC (0.5702), TG (0.5192), and LDL-C (0.4523). The indicators with the highest gain-ratio are TC (0.0724), UA (0.0694), and TG (0.0669), indicating that TC, TG, UA, and LDL-C possess the strongest discriminative power for outcome classification.

**Table 3:** Feature importance ranking.

Feature	Pearson_Rank	*P*-value	InfoGain	Gain_Ratio
DBP	0.1135	0.02	0.1377	0.0238
Hypertension	0.0744	0.12	0.0040	0.0042
Cr	0.0636	0.19	0.2193	0.0350
SBP	0.0625	0.19	0.1596	0.0258
BMI	0.0620	0.20	0.3215	0.0472
UA	0.0567	0.24	0.5389	0.0694
HCT	0.0495	0.30	0.4081	0.0555
RBC	0.0444	0.36	0.4477	0.0594
LDL-C	0.0352	0.47	0.4523	0.0598
ALT	0.0314	0.51	0.1836	0.0307
TBIL	0.0257	0.59	0.3480	0.0480
GGT	0.0256	0.60	0.2538	0.0394
HDL-C	0.0236	0.62	0.1803	0.0289
HB	0.0214	0.66	0.2499	0.0392
PLT	0.0145	0.76	0.4599	0.0625
TC	−0.0051	0.92	0.5702	0.0724
Diabetes	−0.0051	0.92	0.0000	0.0000
HCY	−0.0074	0.88	0.2814	0.0433
Common carotid artery plaque (bilateral/unilateral/non)	−0.0107	0.82	0.0002	0.0001
Age	−0.0137	0.78	0.1166	0.0211
Glucose	−0.0240	0.62	0.2256	0.0386
Gender	−0.0423	0.38	0.0013	0.0019
PDW	−0.0540	0.26	0.1966	0.0311
TG	−0.0879	0.07	0.5192	0.0669
MPV	−0.103	0.03	0.1166	0.0222

DBP, diastolic blood pressure; Cr, creatinine; SBP, systolic blood pressure; BMI, body mass index; UA, uric acid; HCT, hematocrit. RBC, red blood cell; LDL-C, low-density lipoprotein cholesterol; ALT, alanine aminotransferase. TBIL, total bilirubin; GGT, gamma-glutamyl transferase; HDL-C, high-density lipoprotein cholesterol; HB, hemoglobin; PLT, platelet; TC, total cholesterol; HCY, homocysteine; PDW, platelet distribution width; TG, triglyceride; MPV, mean platelet volume.

### Evaluation

We evaluated the predictive performance of five models—LightGBM, XGBoost, SVM, MLP, and Random Forest. The results showed that LightGBM achieved an accuracy of 63.64%, an *F*1-score of 38.89% and an AUC of 49.55%; XGBoost achieved an accuracy of 75%, *F*1-score of 65.60% and an AUC of 62.5%; SVM achieved an accuracy of 63.64%, an *F*1-score of 38.89% and an AUC of 50.00%; MLP achieved an accuracy of 59.09%, an *F*1-score of 37.14% and an AUC of 50.00%; and Random Forest achieved an accuracy of 65.91%, an *F*1-score of 53.09% and an AUC of 56.25%. Overall, XGBoost demonstrated superior predictive performance compared to the other models, as shown in Table 4. Therefore, we conclude that the XGBoost model is effective in predicting different severity levels of OSAHS.

**Table 4:** Performance comparison of different models on the independent test set.

Model	Accuracy (%)	*F*1-score (%)	AUC (%)
LightGBM	63.64	38.89	49.55
SVM	63.64	38.89	50.00
MLP	59.09	37.14	50.00
Random Forest	65.91	53.09	56.25
XGBoost	75.00	65.60	62.50

XGBoost (proposed): best-performing model.

All models were evaluated on the same independent test set. N/A, not applicable.

## Discussion

This study aimed to utilize machine learning technology to develop a clinical index-based model for OSAHS severity stratification. We first collected clinical data from 432 patients at Qujing Central Hospital in Yunnan Province, including 25 feature variables such as demographic information, clinical symptoms, serum biomarkers, and some imaging findings. Then, through correlation analysis and feature importance ranking, we screened out the indicators with the greatest impact on OSAHS severity, including HCY, TBIL, BMI, GGT, and Age. Subsequently, we used these indicators to construct five machine learning models: LightGBM, XGBoost, SVM, MLP, and Random Forest, and employed cross-validation to adjust model parameters to obtain optimal predictive performance. Ultimately, we found that the XGBoost model performed best in predicting OSAHS severity, achieving an accuracy of 75% and an *F*1-score of 65.60 (Liu K. et al., 2024; Somers et al., 2008).

XGBoost is a tree-based algorithm known for its strong data adaptability (Liu M. H. et al., 2024). The XGBoost algorithm was employed for data processing because its decision trees are constructed based on the residuals from the previous tree, which ensures the predictive accuracy of the model (Liu K. et al., 2024). It enables the development of a reliable predictive model for assessing the severity of OSAHS in patients.

OSAHS is a serious sleep disorder characterized by recurrent episodes of upper airway obstruction during sleep, leading to intermittent hypoxia and sleep fragmentation. OSAHS is closely associated with various chronic conditions, such as hypertension, cardiovascular disease, diabetes, and cognitive impairment, posing significant risks to patients' health and quality of life (Punjabi, 2008; Young et al., 2002). However, due to insufficient public awareness of OSAHS, uneven distribution of healthcare resources, and limitations of diagnostic methods, a large number of patients fail to receive timely diagnosis and treatment, leading to deterioration of the condition and increased societal healthcare burden (Wang et al., 2024). Currently, the gold standard for diagnosing OSAHS is PSG. However, the high cost of PSG diagnosis, along with its requirement for specialized equipment and personnel, limits its widespread application in the general population (Somers et al., 2008). Therefore, the development of a rapid, accurate, and easily accessible OSAHS severity stratification model can enable early identification and intervention in high-risk populations. Such a model can assist clinicians in assessing a patient's risk of sleep apnea and determining the need for PSG diagnosis, thereby improving diagnostic efficiency and optimizing the allocation of healthcare resources (Kapur et al., 1999).

The findings of this study hold significant clinical implications. First, this model can serve as a rapid in-hospital baseline screening tool, helping clinicians preliminarily assess a patient's OSAHS risk. It can prioritize patients with moderate-to-severe symptoms for definitive PSG diagnosis, reduce unnecessary examinations for low-risk patients, and thus more effectively concentrate limited medical resources on high-risk populations. Furthermore, the model requires only basic physical examination data, with a single screening cost estimated at less than 10% of that for PSG, which could significantly reduce healthcare expenditures. Second, the model can be applied to community health management and chronic disease management, addressing diagnostic delays caused by the lack of PSG equipment in primary care facilities. This application could improve the early diagnosis and treatment rates for OSAHS. Additionally, the model can provide a reference for personalized treatment, assisting doctors in formulating different treatment plans for patients with varying severity levels. For instance, it could actively recommend non-invasive ventilation therapy for patients with moderate-to-severe OSAHS to prevent serious complications such as cardiovascular and cerebrovascular events. For patients with mild OSAHS, lifestyle interventions or oral appliances could be prioritized. In this context, the XGBoost model's 75% accuracy and 65.6% *F*1-score should be interpreted as a triage tool to prioritize moderate-to-severe patients for PSG, rather than as a standalone diagnostic replacement. Our model enables more direct focus on patients with moderate-to-severe OSAHS, facilitating the provision of optimal treatment strategies at an earlier stage, which is beneficial for patient prognosis and carries substantial clinical significance (Yaggi et al., 2005). The identification of HCY, TBIL, BMI, GGT, and Age as core predictors aligns with the pathophysiology of OSAHS. HCY and TBIL may reflect hypoxia-induced metabolic and endothelial dysfunction, while elevated BMI indicates mechanical upper airway narrowing. Age and GGT are proxies for cumulative metabolic risk.

Despite the positive outcomes of this study, several limitations should be acknowledged. First, the relatively small sample size (*n* = 432) relative to the number of features (25) increases the risk of overfitting, although we mitigated this through feature selection to reduce dimensionality and 5-fold cross-validation on the training set. Second, the moderate AUC values (e.g., 0.625 for XGBoost) warrant specific discussion. Unlike diagnostic models discriminating “patients vs. healthy controls,” our task focuses on differentiating “Mild” from “Moderate-to-Severe” OSAHS within an already diseased population. This intra-group discrimination is inherently challenging due to the pathophysiological overlap between adjacent severity stages. Consequently, we prioritized Accuracy and *F*1-score as the primary metrics, as they directly reflect the model's utility in a clinical triage scenario where misclassification costs are high. Third, as a single-center retrospective study, the generalizability of the model is limited, and external multi-center validation is necessary before clinical deployment. However, the reported Accuracy and *F*1-score on the independent test set provide a pragmatic reflection of the model's performance in a real-world clinical triage scenario. Compared to existing work that primarily relied on demographic and symptom data, our approach explicitly integrates biochemical markers (HCY, TBIL) with routine clinical indicators, offering a more accessible and physiologically relevant screening tool for OSAHS severity prediction. Future research should aim to expand the sample size with multi-center data, refine data collection methods, and explore three-class classification to align with traditional severity grading, thereby enhancing the model's reliability, validity, and clinical granularity. Furthermore, although feature importance analysis was conducted using three complementary methods (Information Gain, Gain Ratio, and Random Forest), advanced explainable AI techniques such as SHAP or LIME were beyond the scope of this initial study and should be incorporated in future work to provide more granular, patient-level interpretability.

ML models hold broad prospects for development. Continued systematic training and multidimensional evaluation are necessary to optimize their predictive performance and improve their feasibility for clinical translation, potentially making them effective auxiliary tools to support physicians in precision diagnosis and treatment.

In summary, this study developed a machine learning model to predict and assess the severity of OSAHS in patients. While it may not entirely replace the current gold standard of PSG, it shows promise as a valuable supplement in clinical practice. Such a tool could serve as an effective triage mechanism for patients with moderate-to-severe OSAHS, potentially improving patient prognosis and creating significant clinical value.

## Data Availability

The raw data supporting the conclusions of this article will be made available by the authors, without undue reservation.
